# Supplementary material for: COZOID: contact zone identifier for visual analysis of protein-protein interactions
Source: BMC Bioinformatics. 2018 Apr 6;19:125. doi: 10.1186/s12859-018-2113-6 (PMC5889581; doi:10.1186/s12859-018-2113-6)
Supplement: Supplementary file 2 — Software build. Executable binary file of the software tool. (ZIP 118,784 kb) [file 12859_2018_2113_MOESM2_ESM.zip › cozoid/platform/docs/testng-6.5.1-javadoc.zip!/org/testng/IConfigurationListener.html]

IConfigurationListener (TestNG)


---


|  |  |  |
| --- | --- | --- |
| **Overview** \| **Package** \| **Class** \| **Tree** \| **Deprecated** \| **Index** \| **Help** | |  |
| **PREV CLASS**   **NEXT CLASS** | **FRAMES**    **NO FRAMES**     **All Classes** |  |
| SUMMARY: NESTED \| FIELD \| CONSTR \| METHOD | DETAIL: FIELD \| CONSTR \| METHOD |  |


---


## org.testng Interface IConfigurationListener

**All Superinterfaces:**: ITestNGListener

**All Known Subinterfaces:**: IConfigurationListener2

**All Known Implementing Classes:**: TestListenerAdapter, TestNG.ExitCodeListener

---

``` public interface IConfigurationListener extends ITestNGListener ```

Listener interface for events related to configuration methods.

---

| **Method Summary** | |
| --- | --- |
| `void` | `onConfigurationFailure(ITestResult itr)`             Invoked whenever a configuration method failed. |
| `void` | `onConfigurationSkip(ITestResult itr)`             Invoked whenever a configuration method was skipped. |
| `void` | `onConfigurationSuccess(ITestResult itr)`             Invoked whenever a configuration method succeeded. |

**Method Detail**

### onConfigurationSuccess

```
void onConfigurationSuccess(ITestResult itr)
```

:   Invoked whenever a configuration method succeeded.

---


### onConfigurationFailure

```
void onConfigurationFailure(ITestResult itr)
```

:   Invoked whenever a configuration method failed.

---


### onConfigurationSkip

```
void onConfigurationSkip(ITestResult itr)
```

:   Invoked whenever a configuration method was skipped.


---


|  |  |  |
| --- | --- | --- |
| **Overview** \| **Package** \| **Class** \| **Tree** \| **Deprecated** \| **Index** \| **Help** | |  |
| **PREV CLASS**   **NEXT CLASS** | **FRAMES**    **NO FRAMES**     **All Classes** |  |
| SUMMARY: NESTED \| FIELD \| CONSTR \| METHOD | DETAIL: FIELD \| CONSTR \| METHOD |  |


---
